# Melatonin Secretion and Impacts of Training and Match Schedules on Sleep Quality, Recovery, and Circadian Rhythms in Young Professional Football Players

**DOI:** 10.3390/biom15050700

**Published:** 2025-05-11

**Authors:** Antonio Almendros-Ruiz, Javier Conde-Pipó, Paula Aranda-Martínez, Jesús Olivares-Jabalera, Darío Acuña-Castroviejo, Bernardo Requena, José Fernández-Martínez, Miguel Mariscal-Arcas

**Affiliations:** 1 Health Science and Nutrition Research (HSNR-CTS1118), Department of Nutrition and Food Science, School of Pharmacy, University of Granada, 18071 Granada, Spain; aalmendros@correo.ugr.es (A.A.-R.); jconde@ujaen.es (J.C.-P.); 2 FSI Lab, Football Science Institute, 18016 Granada, Spain; jesusyolivares@gmail.com (J.O.-J.); bernardorequena@fsi.training (B.R.); 3 Department of Health Sciences, Faculty of Health Sciences, University of Jaén, 23071 Jaén, Spain; 4 Centro de Investigación Biomédica, Facultad de Medicina, Departamento de Fisiología, Instituto de Biotecnología, Parque Tecnológico de Ciencias de la Salud, Universidad de Granada, 18016 Granada, Spain; ampaula@correo.ugr.es (P.A.-M.); dacuna@ugr.es (D.A.-C.); 5 Instituto de Investigación Biosanitaria de Granada (ibs.GRANADA), 18016 Granada, Spain

**Keywords:** circadian rhythm, melatonin, training time, football performance, sleep

## Abstract

Modern elite football is becoming increasingly physically demanding, often requiring training and matches to be played at night. This schedule may disrupt circadian rhythms and melatonin secretion, thereby impairing sleep and recovery. This study investigated the effects of training time on melatonin secretion, circadian phase markers, and sleep parameters in elite youth soccer players. Forty male players (aged 16–18 years) from an elite Spanish youth football club were studied. Two groups followed the same training program but trained either in the morning (MT) or in the evening (ET). Salivary melatonin was measured at six time points to determine the mean levels, dim light melatonin onset (DLMO), amplitude, and acrophase. Chronotype, sleep quality (PSQI), and daytime sleepiness (ESS) were assessed using validated questionnaires. Dietary intake and anthropometric variables were also recorded. The MT group had higher mean melatonin levels (*p* = 0.026) and earlier DLMO (*p* = 0.023) compared to the ET group. Sleep quality was significantly better in the MT group (*p* < 0.001), despite shorter sleep duration (*p* = 0.014). No major differences in diet or anthropometry were observed. The chronotype had a secondary effect on the circadian markers. Evening training is associated with alterations in melatonin rhythms and reduced sleep quality, possibly due to light-induced chronodisruption. These findings highlight the importance of training timing as a modifiable factor in the chronobiology and recovery of athletes. Incorporating circadian principles into training schedules may optimize resting time and thus performance and long-term health in athletes.

## 1. Introduction

Elite football (also known as soccer) is a complex sport characterized as an intermittent team sport, alternating bouts of high-intensity actions with longer periods of low activity [1]. Since 2006, the number of matches and training sessions per season has increased, with shorter recovery periods between them [2]. Furthermore, the average number of high-intensity actions per match has increased by up to 50% [1], making modern football more physically demanding [3] and increasing the risk of injury. In this context, rest and sleep have become crucial components of football players’ physical conditioning and performance optimization [4]. Insufficient sleep duration and quality have negative effects on athletic performance and can negatively impact the recovery process after a match, affecting muscle glycogen replenishment, delaying muscle repair, impairing cognitive function, and increasing mental fatigue [5,6]. However, several factors can disrupt sleep and recovery, including irregular match schedules, travel-related fatigue, and prolonged exposure to artificial light [5]. In this regard, environmental conditions, such as bright light in the stadium or light emitted from the screens, can significantly impact sleep quality by disrupting circadian rhythms and melatonin production, leading to sleep disturbances and misalignment [7,8,9]. The biological clock, located in the suprachiasmatic nuclei (SCN) of the hypothalamus and composed of approximately 20,000 neurons, regulates circadian rhythms through the expression of clock genes in response to the light–dark cycle [10]. It also controls melatonin production, a tryptophan-derived hormone secreted by the pineal gland, which peaks at night and is suppressed by light during the day [11]. Melatonin, in turn, coordinates the sleep–wake cycle and other biological processes with the light–dark cycle, making it a reliable marker of sleep quality and phase shifts in the sleep–wake cycle [12]. On the other hand, each individual has a biological preference for activity and rest, known as a chronotype [13]. People can be classified into three chronotypes: morning-type, who perform better in the morning; evening-type, who perform better in the evening; and neither-type, who do not show a strong preference for either [14]. However, the impact of chronotype on sleep quality and its relationship with sports performance remains unclear [15]. In professional football players, studies have shown that training or playing in the evening compared to during the day is associated with poorer sleep quality and fewer hours of rest [5,16]. However, the evidence supporting these perceptions, particularly concerning night-time training, matches, and the influence of melatonin levels, remains inconclusive, with this aspect being important to emphasize as a gap in the current literature that this study aims to fill [17,18]. In this context, the present study aims to investigate the impact of training schedules on sleep quality, recovery perception, and circadian rhythms in young professional football players. Specifically, the study seeks to determine whether training in the evening affects melatonin secretion and sleep duration compared to daytime sessions. Additionally, this study will explore the role of chronotype. Given the growing demands of modern football, understanding these interactions is crucial for optimizing training schedules, enhancing recovery strategies, and ultimately improving player performance and well-being.

## 2. Materials and Methods

### 2.1. Study Design and Ethics

This descriptive, comparative, cross-sectional, and non-experimental study examined the effect of training at different times of day on circadian markers and sleep quality in professional male football players under 19 years of age. The participants were drawn from the first division of Spanish youth football, the highest competitive level for this age group. This study compared players with similar characteristics and identical training programs, differing only in their training schedules (morning training (MT) vs. evening training (ET)), using both objective and subjective markers (melatonin rhythm, chronotype, sleep quality, and daytime sleepiness). To control for potential environmental influences on melatonin production, the light intensity was measured at each team’s training location during their respective training times using a lux meter. The MT group was exposed to natural sunlight, while the ET group trained under artificial lighting due to visibility constraints. The light intensity values obtained (53,626 lux for the MT group and 4154 lux for the ET group) are known to suppress melatonin production, with studies showing that exposure to light intensities of between 200 and 400 lux for less than two hours can cause significant suppression [19,20,21]. This is especially relevant for the ET group, as melatonin naturally starts to be produced later in the day. Additionally, we also performed anthropometric and nutritional assessment measurements by training group to account for any differences between the groups. All study protocols and procedures were conducted in accordance with the Declaration of Helsinki and approved by the Ethics Committee of the University of Granada (protocol code 3340/CEIH/2023, 18 May 2023 and PPJIB2021-22, 16 May 2022). The players were fully informed about the study’s objectives and methods, and each provided written informed consent before participation. For underage players, legal guardians were informed, and they authorized the minor’s participation through the corresponding informed consent.

### 2.2. Participants

Two youth-category teams from the same Spanish club participated in the study, with a total of 40 players: 17 in the MT team (age 17. 59 ± 0.62 years) and 23 in the ET team (age 16.61 ± 0.58 years). The inclusion criteria were as follows: (a) being in good health and possessing medical clearance for federated sports; (b) belonging to one of the two youth-category teams in the Spanish league; (c) being a federated football player; (d) training either in the morning (9:00–10:30 a.m.) or in the evening (20:00–21:30 p.m.); and (e) undergoing measurements in March during the competitive phase. The exclusion criteria were (a) not being within the eligible youth-category age range of 16–18 years and (b) taking melatonin supplements.

### 2.3. Procedures

Evaluations were conducted during each team’s training schedule. On the day of evaluation, the players were instructed to refrain from high-intensity exercise, training, or stretching. Consequently, anthropometric measurements were taken before training. Specifically, the anthropometric measurements were taken on the same day as the melatonin sampling began but before any training activities. The players were instructed to avoid intense physical activity for at least 24 h prior to saliva sampling. No training was conducted on the saliva collection day, and training was resumed the following day. All the players were already familiar with the procedures, as they routinely performed them as part of their usual activities. Afterward, the players were instructed on how to autonomously collect saliva samples and were provided with the necessary materials. The saliva collection took place midweek (between Wednesday and Thursday) to allow at least 72 h of recovery after the higher-intensity weekend match. The samples were then returned to the club and sent to the laboratory for analysis. The various sleep-related and nutritional questionnaires were explained beforehand, and the players completed them during the same session. Any doubts were addressed to ensure clarity and standardization in their completion.

### 2.4. The Measurement of the Circadian Rhythm of Melatonin

Saliva was used to determine melatonin levels, as it is a non-invasive method that correlates well with blood melatonin levels [22,23]. The participants were instructed to not eat or drink for 30 min before sample collection and to avoid exposure to blue light during night-time collections. The participants were given a verbal and written explanation of what constitutes blue light, including examples of common sources, such as smartphones, tablets, televisions, and LED lighting. They were specifically instructed to avoid screens and bright artificial lights for at least one hour before and during sample collection, especially for night-time samples. The saliva was self-collected using Salivettes^®^ (Sarstedt AG & Co. KG, Sarstedtstrabe, Nümbrecht, Germany), which contain a neutral cotton swab, at six different time points: 4 and 2 h before bedtime, at bedtime, 2 and 4 h after bedtime, and upon waking. The date and season of collection were recorded. To preserve the sample integrity, the participants were instructed to refrigerate the daytime samples, covered with aluminum foil, until shipment. The night-time samples could be kept at room temperature until the following morning. All the samples were then placed in a padded envelope and returned to the club, from where they were sent to the laboratory via a refrigerated courier service to ensure delivery within 24 h. Upon arrival, the samples were centrifuged, pipetted, and stored at −80 °C until the analysis. The saliva samples were analyzed at the Centro de Investigación Biomédica of the University of Granada, following a method established by our group and previously used in a study with prostate cancer patients [24]. Briefly, the samples were thawed, and melatonin was extracted using a liquid–liquid method with chloroform. For each 250 μL saliva sample, 1 mL of chloroform was added to glass vials, then shaken for 10 min at 2500 rpm and centrifuged at 3900 rpm for 10 min. The organic phase (800 μL) was transferred to new vials, and chloroform was evaporated under a vacuum using a SPD2010 Savant SpeedVac (ThermoFisher Scientific S.L., Alcobendas, Madrid, Spain). The residues were reconstituted in 100 μL of 5% acetonitrile, shaken, and then injected into the UltiMate 3000 UHPLC system (ThermoFisher Scientific S.L., Alcobendas, Madrid, Spain) for chromatographic separation. A volume of 25 μL of the sample was injected into a reverse-phase Hypersil GOLD C18 column (2.1 mm × 100 mm, 1.9 μm) with a mobile phase of 0.1% formic acid in water (A) and acetonitrile (B) at a flow rate of 0.4 mL/min. A linear gradient was applied from 5% to 98% B over 7 min, held at 98% for 2.1 min, and equilibrated for 0.9 min back to 5% B. The total analysis time was 10 min. The autosampler and column oven were set at 10 °C and 45 °C, respectively. Melatonin was detected using an Orbitrap Q-Exactive Focus mass spectrometer (ThermoFisher Scientific S.L., Alcobendas, Madrid, Spain) in positive electrospray ionization (ESI) mode, with selective ion monitoring (SIM) for the protonated melatonin ion (*m*/*z* 233.12845) at a retention time of 3.98 min. Melatonin concentrations were quantified using an external calibration curve. Any subjects with undetectable or excessively high melatonin levels were excluded from the analysis. The average melatonin level in pg/mL, amplitude (maximum melatonin peak) in pg/mL, acrophase (time of maximum melatonin peak) in decimal hours, and dim light melatonin onset (DLMO) in decimal hours were determined. The average melatonin circadian rhythm was represented for each group and further stratified by chronotype. The median of the MT group was used as the cutoff to categorize low or high average melatonin levels and amplitudes. The acrophase and DMLO were classified as early or late using the same criteria [24].

### 2.5. Questionnaires Related to Sleep

Chronotypes were determined using the Horne and Östberg Morningness–Eveningness Questionnaire [25], consisting of 19 questions about morning and evening habits, with a total score ranging from 16 to 86 points. Based on the total score, the participants were classified into the following chronotypes: definitely evening type (16–30 points), moderately evening type (31–41 points), neither type (42–58 points), moderately morning type (59–69 points), and definitely morning type (70–86 points). From this, three chronotypes were determined in our study: (1) moderately evening type (ME), (2) neither type (N), and (3) moderately morning type (MM).

Sleep duration and quality were evaluated using the Pittsburgh Sleep Quality Index (PSQI) [26], based on sleep habits during the month prior to the interview. Sleep duration was calculated by subtracting the sleep onset from the sleep end. The PSQI includes 19 questions grouped into seven components: subjective sleep quality, sleep latency, sleep duration, sleep efficiency, sleep disturbances, the use of medication, and daytime dysfunction, each scored from 0 to 3 points. The total score (0–21) indicates the overall sleep quality, with higher scores reflecting greater difficulty. The participants were classified as having good (≤5 points) or poor sleep quality (>5 points). Daytime sleepiness was assessed using the Epworth Sleepiness Scale (ESS) [27], which measures the likelihood of dozing or falling asleep in various everyday situations. The ESS consists of eight items scored on a scale from 0 (no risk of dozing) to 3 (a high risk of dozing), resulting in a total score ranging from 0 to 24, with higher scores indicating greater daytime sleepiness. The participants were classified based on their total score: a score of ≤9 was considered as normal sleepiness (low-risk sleepiness, LR), whereas a score of ≥10 indicated excessive daytime sleepiness (high-risk sleepiness, HR), possibly indicating a pathological condition.

### 2.6. Anthropometric Measures

Anthropometric data were gathered following ISO 7250-1:2017 guidelines [28] and the standards of the International Society for the Advancement of Kinanthropometry (ISAK) [29]. The measurements included two basic parameters (body mass and height), six skinfold thicknesses (triceps, subscapular, supra-iliac, abdominal, thigh, and calf), and three circumferences (relaxed arm, waist, and hip). A CESCORF inextensible metal tape (CESCORF, Tristeza, Porto Alegre/RS, Brazil) was employed to measure the circumferences, and a Holtain mechanical caliper (HOL-98610ND) with 0.2 mm precision was used for the skinfold assessments. Each anthropometric measurement was taken two or three times. Specifically, when the technical measurement error between the first two measurements surpassed 5% for skinfolds or 1% for the other measurements, a third measurement was performed. The mean or median average was then calculated, as appropriate, for subsequent analyses. This protocol helps ensure precision and consistency in the data, thereby reinforcing the validity of the research findings and facilitating a more accurate interpretation of any differences observed. The room temperature was standardized at 24 °C during all the measurements. The Faulkner formula [30] was used to calculate the fat mass as a percentage. Two health indicators were also derived: the waist-to-hip ratio and the body mass index (BMI).

### 2.7. Dietary Assessment

A 24 h recall questionnaire (R24h) was employed to retrospectively assess and quantify food and beverage intake during the 24 h prior to the interview [31,32]. This method enables the estimation of the energy and nutrient content of the subjects’ diets. On this occasion, each participant completed three 24 h recalls—two on weekdays and one on a weekend day—to enhance the accuracy. Experienced interviewers assisted in administering the questionnaire to improve the data quality and minimize any recall bias. The DIAL software (version 3.15, 2021, Alce Ingenieria, Madrid, Spain) was used to process the R24 h results, translating the reported food items into nutrient profiles for further analysis. The basal metabolic rate (BMR) was calculated using the Harris–Benedict formula [33]. The total energy expenditure (TEE) was subsequently estimated in accordance with the method proposed by the FAO, WHO, and the United Nations University [34], which uses the BMR as its basis. A physical activity level (PAL) factor of 2.15—appropriate for men performing heavy physical activity—was applied to determinate the (TEE) estimate.

### 2.8. Statistical Analysis

In the descriptive analysis, the mean and standard deviation were calculated for the continuous quantitative variables, and percentages were determined for the categorical variables, in order to compare characteristics between the MT and ET groups. The categorical variables were examined using either the Chi-square or Fisher’s exact tests to determine the significance of observed differences, whereas continuous variables were analyzed with the Mann–Whitney U test or Student’s *t*-test, as appropriate. Additionally, the circadian rhythm of melatonin was plotted for both groups—morning and evening—and further stratified according to chronotype. Effect sizes were calculated to complement the interpretation of the statistical analyses. For the continuous variables, Cohen’s d with 95% confidence intervals was computed, where values of 0.2, 0.5, and 0.8 were interpreted as small, moderate, and large effects, respectively. For the categorical variables, Cramér’s V was used to assess the strength of association, with thresholds of 0.1 (small effect), 0.3 (moderate effect), and 0.5 (large effect). All the statistical tests were performed as two-sided, and the statistical significance was defined at *p* < 0.05. The analyses were conducted using R version 4.0.2 (R Foundation for Statistical Computing, Vienna, Austria), along with ggplot2 version 3.4.2.

## 3. Results

### 3.1. Anthropometric Characteristics

A total of 40 the players, 17 in the morning training group and 23 in the evening training group, were evaluated. Table 1 presents the anthropometric measurements by training group. No significant differences were observed in weight, height, BMI, or most skinfold thicknesses. The subscapular skinfold showed a borderline non-significant difference between the groups (*p* = 0.051). In contrast, the suprailiac skinfold thickness was significantly higher in the morning training group compared to the evening training group (30.95 ± 1.54 mm vs. 28.91 ± 1.68 mm; *p* = 0.002).

### 3.2. Daily Energy and Nutrient Intake

As shown in Table 2, the BMR and TEE were similar between the morning and evening training groups (*p* = 0.448). Similarly, there were no significant differences in the total energy intake (EI), water intake, or macronutrient intake (protein, carbohydrates, and lipids), although the clinical significance is important because of its similarity to the methodological recommendations to estimates for these population groups [32,33,34]. The proportions of energy from proteins, carbohydrates, and lipids were also similar (all *p* > 0.30), and no significant differences were found in cholesterol or fiber intakes.

### 3.3. Melatonin Profile and Circadian Parameters

Table 3 shows the differences in the melatonin level, amplitude, DLMO, and acrophase between the two training groups. The MT group showed a higher mean melatonin concentration (52.49 ± 41.81 vs. 29.93 ± 21.19 pg/mL; *p* = 0.026), with a moderate-to-large effect size (Cohen’s d = 0. 68; CI: 0.06–1.36). A post hoc power analysis was conducted based on the observed effect size for melatonin concentrations. Given the available sample size, the achieved statistical power was 67% (α = 0.05, two-tailed). Although slightly below the conventional 80% threshold, this level of power is considered acceptable given the moderate-to-large effect size observed.

Similarly, the DLMO was also significantly earlier in the MT group (21.20 ± 1.36 vs. 21.93 ± 1.48 h; *p* = 0.023), with a greater proportion of individuals exhibiting an early DLMO (<20.5 h) than in the ET group (41.1% vs. 8.7%; *p* = 0.040). No significant differences were observed in melatonin amplitude or acrophase between the groups.

**Figure 1:**
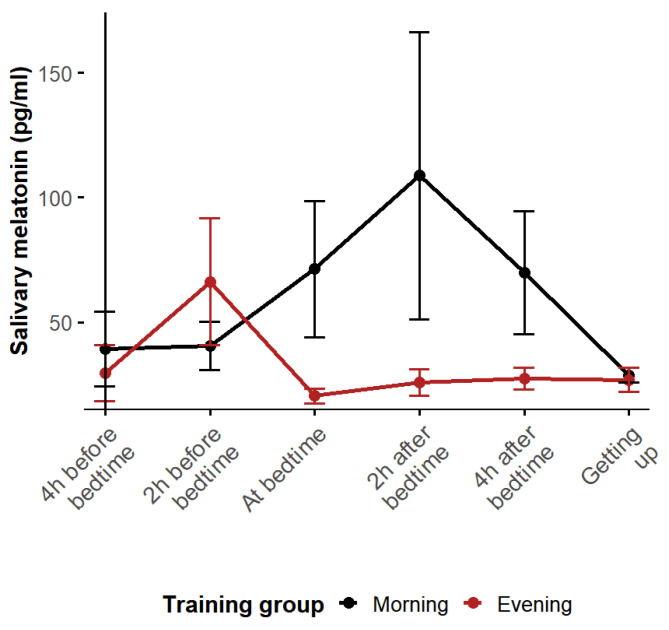
Average observed melatonin rhythms of training groups.

The average melatonin levels were consistently lower in the ET group than in the MT group at almost all time points, except 2 h before bedtime (around 22:30 p.m. for the ET group), which corresponds to the ET group’s acrophase (Figure 1), indicating a phase advance. Furthermore, this group showed a steeper decline in melatonin levels compared to the MT group, possibly due to the inhibition of melatonin caused by light exposure during night-time training. In contrast, the MT group exhibited a normal melatonin rhythm, with a typical acrophase occurring after bedtime (around 2:15 a.m. for the MT group).

**Figure 2:**
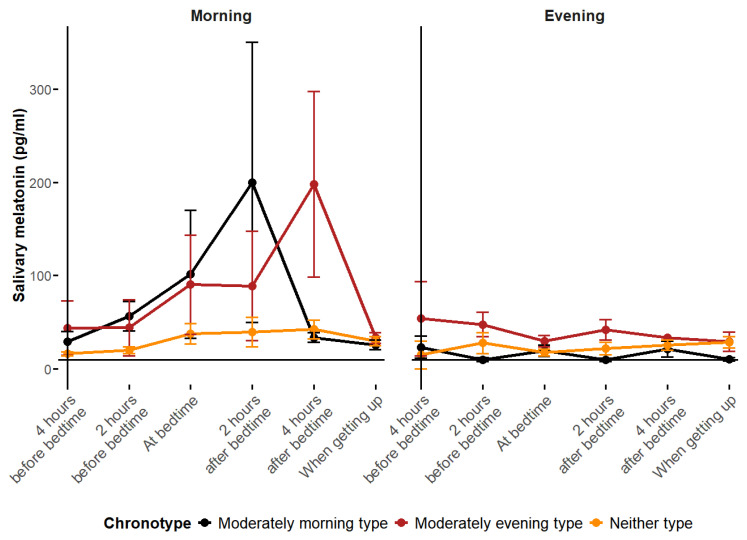
Average observed melatonin rhythms by chronotype.

When comparing average salivary melatonin rhythms by both training group and chronotype, the differences between the training groups become even more pronounced (Figure 2). In the MT group, the players with the MM and ME chronotypes exhibited higher average melatonin levels compared to those with the N chronotype. In the ET group, the players with the ME chronotype had higher average melatonin levels than the other chronotypes. Regarding circadian melatonin rhythms, all the chronotypes in the MT group showed a defined acrophase, which aligned well with their respective chronotypes. In contrast, the ET group exhibited a complete loss of the melatonin circadian rhythm across all the chronotypes.

### 3.4. Sleep Characteristics

Table 4 summarizes the sleep-related variables for both groups. The participants in the MT group woke up significantly earlier (7.22 ± 0.40 vs. 8.58 ± 1.54 h; *p* < 0.001), slept fewer total hours (6.95 ± 0.40 vs. 7.95 ± 1.31 h; *p* = 0.014), and reported good sleep quality more frequently than the ET group (70.6% vs. 47.8%; *p* < 0.001). The chronotype distribution also differed between the groups, with a higher proportion of ME in the ET group (75.1% vs. 47.1%; *p* < 0.001) and a lower proportion of MM and N (16.6% vs. 35.3% and 8.3% vs. 17.6%, respectively; *p* < 0.001). Daytime sleepiness did not differ significantly between the groups (*p* = 0.678).

**Figure 3:**
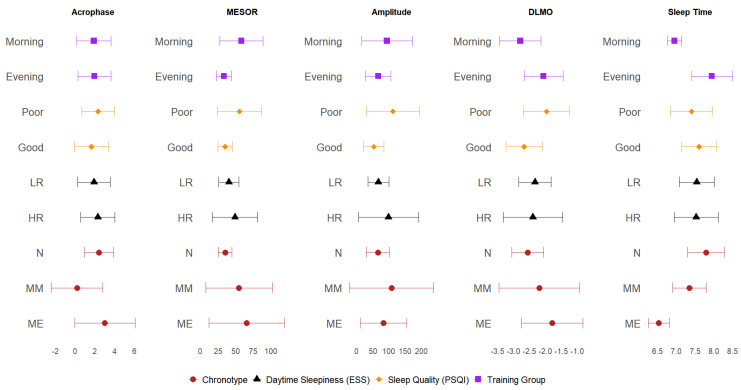
Comparison of mean values for acrophase, MESOR, amplitude, DLMO, and total sleep time by training group, chronotype, ESS, and PSQI.

Figure 3 shows a comparison of means values and 95% confidence intervals for the acrophase, circadian melatonin mean (MESOR), amplitude, DLMO, and total sleep time by the training group, sleep quality, daytime sleepiness risk, and chronotype. There were notable differences between these subgroups, particularly in the training group, sleep quality, and chronotype. Notably, the sleep duration was the shortest in the morning training group and among individuals with a morning chronotype. Those players with good sleep quality had an early acrophase and DMLO, but a lower MESOR and amplitude.

## 4. Discussion

The present study analyzed the impact of training time on circadian rhythms and sleep quality in elite youth football the players. The findings suggest that training in the evening is associated with lower melatonin levels, a delayed DLMO, and reduced sleep quality compared to morning training. These results provide further evidence to support the hypothesis that the alteration of training schedules and the subsequent exposure to artificial light can disrupt circadian rhythms and sleep, potentially affecting recovery and performance and thus increasing the risk of injury.

Although previous studies have demonstrated that anthropometric characteristics, body composition, and dietary patterns can influence circadian rhythms, melatonin levels, and sleep quality [12,35], these variables did not differ significantly between the morning and evening training groups in our study. Therefore, it is unlikely that these factors contributed to the observed differences between groups. This finding reinforces the assumption that the training time itself is a critical factor affecting circadian rhythms, melatonin secretion, and sleep-related outcomes.

As we hypothesized, the MT group had higher mean salivary melatonin levels and also an earlier DLMO than the ET group, suggesting a more synchronized circadian rhythm. Although studies specifically examining the relationship between the training time, circadian rhythm, and sleep quality are scarce, these results are in line with similar studies carried out in shift workers [36]. However, the MT group unexpectedly had a shorter sleep duration but reported better sleep quality than the ET group. This contrasts with other studies carried out on elite football players [16] and basketball players [7], which reported a longer sleep duration in the morning training group. While this result may seem surprising, it could be partially explained by contextual factors, such as the time of their first training or academic activities in the morning, which may have led to an earlier wake-up time. Sargent et al. [37] highlight that the timing of training can affect the sleep duration, with early wake-ups often leading to a shorter sleep duration and increased levels of pre-training fatigue in elite athletes. Notably, despite the shorter sleep duration, the sleep quality was perceived as better in the MT group. This may be linked to an earlier melatonin onset and sustained higher levels throughout the sleep cycle, suggesting that the MT group required less sleep to feel recovered. On the other hand, the sleep time reported in other studies that focus on football the players were more similar to the ET group (>8 h) [38]. It is possible that the MT group, who have more time to rest after training, spent more time on the social media before bedtime, a factor that has been associated with up to one hour less sleep in professional football players [5,39]. Although the subjective sleep quality was higher in the MT group, the shorter sleep duration observed may still have implications for recovery and performance. Previous research has shown that sleep restriction negatively impacts both physical and cognitive performance in athletes [40,41]. These findings suggest that sleep strategies for athletes should balance both the sleep quality and duration to optimize recovery and performance.

Thus, the explanation for our results can be found in the influence of light exposure, which is the primary external factor regulating melatonin secretion [42,43]. In this context, playing football in the evening may have a chronodisruptive effect on sleep, as it involves prolonged exposure to bright artificial light in the stadium, especially when matches are scheduled close to bedtime, as commonly occurs in elite European competitions, such as the Champions League. This is supported by Kneizer et al. [44], who found that bright light may reduce melatonin levels but not athletic performance. Conversely, several studies reviewed by Nedelec et al. [5] have reported no negative effects or even beneficial outcomes on sleep following night-time exercise. The authors of these studies suggest that differences in exercise intensity might explain these findings, as the sports analyzed were generally less physically demanding and stressful compared to football. Nevertheless, other studies have reported phase-delaying effects on melatonin rhythms when exercise was performed at night, regardless of whether it took place in darkness or under very dim lighting conditions [43]. From a practical standpoint, with the data from this study, coaches might consider adjusting training schedules based on the players’ chronotypes, considering morning training to support better melatonin regulation, and exploring interventions such as morning light exposure or blue-light blocking strategies in the evening, as indicated by studies of practical guidelines for improving sleep and performance in athletes [45].

Regarding chronotype, although its analysis was a secondary objective of this study, we observed some differences. When analyzing the training groups separately, the melatonin curves were similar among the chronotypes MM and ME. In the morning group, the MM chronotype exhibited a slight phase advance in the melatonin curve, while in the evening group, the ME chronotype showed a slight phase delay in the melatonin curve. When considering the sample as a whole, the MM chronotype displayed an earlier DLMO and a higher average sleep duration compared to the ME chronotype. Therefore, when comparing morning and evening training, chronotype appears to have a secondary influence on circadian rhythms, suggesting that the training time may be a more determining factor; when comparing between chronotypes, the MM individuals sleep better than ME individuals. In any case, and in line with other research [46,47], the ME chronotype may benefit from phase advances induced by morning exercise, while evening exercise may exacerbate circadian misalignment in the MM chronotype, and this should be taken into account when scheduling training sessions [14].

### Limitations

This study has several limitations that should be considered when interpreting the results. The cross-sectional design prevents establishing causal relationships between the training time, melatonin secretion, and sleep quality, highlighting the need for longitudinal studies to assess long-term adaptations. Another important limitation of this study is the imbalance in group sizes between the morning and evening groups. This imbalance may have reduced the statistical power and affected the stability of the effect size estimates. Although a post hoc power analysis suggested an acceptable power for the main comparison, future studies should aim for balanced group sizes to improve the internal validity. Furthermore, the study sample was composed of elite youth athletes from the same team, which limited the number of available participants. While this enhances sample homogeneity and control, it may reduce the generalizability of the results to other athletic populations. Additionally, the sleep parameters were assessed using self-reported questionnaires, which may introduce a recall bias. The absence of objective performance assessments limits our ability to determine whether disrupted circadian rhythms and reduced sleep quality directly translate into performance impairments. In addition, including a recovery perception scale, such as the Total Quality Recovery (TQR) scale, would have enriched the interpretation of the sleep and melatonin data. While environmental and lifestyle factors, such as diet and training load, were controlled, other variables, including screen exposure before bedtime, stress, and individual sleep habits, may have influenced the results. In addition, the absence of any formal control for caffeine intake or psychological stress may have introduced variability into the melatonin secretion and sleep outcomes. Future studies should incorporate standardized screening for caffeine intake and validated tools for assessing stress (e.g., PSS or RESTQ-Sport). Furthermore, although the artificial lighting at training locations was measured, individual variations in light exposure outside training were not considered, which could have impacted melatonin secretion and sleep patterns. In these future studies, the target population should also be instructed about the use of social media [39] and screens before going to bed in the case of experimental studies. Lastly, core body temperature, a key factor in sleep onset and recovery, was not measured, warranting further investigation to understand its role in training-related circadian disruptions. Despite these limitations, this study provides valuable insights into the influence of training schedules on circadian rhythms and sleep quality in elite youth football the players, emphasizing the need for further research in this area.

## 5. Conclusions

The strengths of our study, and a novelty in the study of circadian rhythms and sleep in professional youth football the players, is that we used salivary melatonin as an objective marker.

This study provides evidence that training time significantly influences circadian rhythms and sleep quality in elite youth football players. The players who trained in the morning had higher melatonin levels, an earlier DLMO, a better-aligned melatonin circadian rhythm, and better sleep quality compared to those who trained in the evening. These findings suggest that exposure to artificial lighting during night-time training sessions may have a chronodisruptive effect, delaying melatonin secretion and impairing sleep parameters.

Despite the fact the evening training group reported a longer sleep duration, their sleep quality was poorer, reinforcing the idea that the total sleep time alone is not a sufficient indicator of recovery. In addition, differences in melatonin secretion and sleep parameters were observed between chronotypes, with the morning-type players generally showing an earlier DLMO and a longer sleep duration. However, chronotype appeared to play a secondary role, with the training time being a more influential factor in determining circadian and sleep-related outcomes.

These findings highlight the importance of considering training schedules when planning elite football training programs. Coaches might consider adjusting training schedules based on the players’ chronotypes, considering morning training to support better melatonin regulation, and exploring interventions such as morning light exposure or blue-light blocking strategies in the evening, as evening sessions may disrupt circadian alignment and potentially affect recovery and performance.

Future research should further explore the physiological and performance-related consequences of night-time training and competition, including the role of meal timing, changes in core body temperature, and individualized strategies to mitigate the negative effects of late-night activity on sleep and circadian rhythms.

## Figures and Tables

**Table 1:** Anthropometric characteristics of the study sample by training group.

Variable (Mean, SD)	Training Group		Sig.
	Morning (*n* = 17)	Evening (*n* = 23)	*p* ^†^
Weight (kg)	70.74 (5.19)	71.31 (6.33)	0.328
Height (cm)	176.29 (5.68)	179.15 (5.83)	0.097
BMI (kg/m^2^)	22.74 (0.79)	22.20 (1.50)	0.183
Faulkner Fat (%)	11.78 (1.34)	13.09 (2.99)	0.335
Tricipital skinfold (mm)	8.06 (1.81)	7.79 (1.56)	0.690
Subscapular skinfold (mm)	9.02 (1.17)	8.30 (1.59)	0.051
Abdominal skinfold (mm)	10.44 (3.53)	10.72 (4.01)	0.798
Bicipital skinfold (mm)	10.59 (2.33)	10.53 (2.65)	0.744
Calf skinfold (mm)	6.41 (1.26)	6.35 (1.34)	0.831
Thigh skinfold (mm)	11.17 (4.13)	10.22 (2.51)	0.864
Suprailiac skinfold (mm)	30.95 (1.54)	28.91 (1.68)	**0.002**
Waist (cm)	75.83 (3.18)	75.68 (3.51)	1.000
Hip (cm)	93.74 (3.30)	93.24 (3.68)	0.745
Arm, relaxed (cm)	30.95 (1.54)	28.91 (1.68)	0.685
Waist-to-hip ratio (WHR)	0.81 (0.03)	0.81 (0.03)	0.901

Notes: ^†^ Student’s *t* and Mann–Whitney U tests were used to calculate differences between training groups. Values are presented as mean ± standard deviation. SD: standard deviation; BMI: body mass index. Values in bold are statistically significant (*p* < 0.05).

**Table 2:** Macronutrients daily intake by training group.

Variable	Training Group		Sig.
	Morning	Evening	*p* ^†^
BMR, kcal *	1881.95 (90.02)	1836.29 (295.22)	0.448
TEE, kcal *	4046.20 (193.55)	3948.02 (634.71)	0.448
Energy intake, kcal *	3024.87 (550.24)	3203.71 (521.87)	0.300
Energy intake/TEE	0.66(0.28)	0.45(0.42)	0.339
Water, g *	3052.76 (1201.98)	2655.63 (1061.57)	0.420
Protein, g *	149.92 (32.01)	154.90 (33.50)	0.692
Carbohydrate, g *	303.49 (86.97)	322.06 (84.43)	0.872
Lipid, g *	124.86 (20.61)	134.14 (28.66)	0.322
Simple carbohydrate			
Glucose, g *	12.85 (8.19)	17.07 (12.40)	0.322
Fructose, g *	15.74 (11.93)	23.74 (28.58)	0.369
Lactose, g *	7.54 (10.60)	6.39 (10.69)	0.547
Soluble fiber, g *	9.22 (3.13)	9.13 (3.89)	0.836
Indissoluble fiber, g *	15.46 (6.26)	13.99 (5.16)	0.565
Cholesterol, mg *	503.47 (187.22)	581.40 (224.99)	0.369
Caloric profile			
Proteins, %	20.29 (5.45)	19.25 (2.47)	0.982
Carbohydrates, %	39.48 (6.83)	40.17 (7.49)	0.982
Lipids, %	37.41 (3.72)	37.82 (6.37)	0.982
Lipids profile			
SFA, %	31.57 (7.25)	35.74 (11.84)	0.534
MUFA, %	62.12 (10.29)	65.20 (11.85)	0.433
PUFA, %	19.11 (5.58)	20.20 (9.90)	1.000

Notes: * Mean (SD): ^†^ Student’s *t* and Mann–Whitney U tests were used to calculate differences between training groups. BMR: basal metabolic rate; TEE: total energy expenditure; SFA: saturated fatty acids; MUFA: monounsaturated fatty acids; PUFA: polyunsaturated fatty acids.

**Table 3:** Association between melatonin level, amplitude, DLMO, and acrophase with training group.

Variable	Training Group		Effect Size	Sig.
	Morning	Evening	(95% CI)	*p*
Melatonin, pg/mL:				
Mean (SD)	52.49 (41.81)	29.93 (21.19)	*d* = 0.71 (0.06, 1.36)	0.026 *
Median	31.6	24.2		
p25; p75	23.7; 69.2	17.8; 31.7		
N° Melatonin (n, %)				
Low (<31.6 pg/mL)	8 (47.1)	17 (69.6)	V = 0.275	0.267 ^†^
High (>31.6 pg/mL)	9 (52.9)	6 (30.4)		
Amplitude, pg/mL:				
Mean (SD)	102.6 (164.7)	66.6 (94.9)	*d* = 0.34 (−0.29, 0.97)	0.311 *
Median	38.9	26.5		
p25; p75	23.7; 108.0	14.1; 63.4		
N° Amplitude (n, %)				
Low (<38.9 pg/mL)	8 (47.1)	13 (56.5)	V = 0.095	0.893 ^†^
High (>38.9 pg/mL)	9(52.9)	10 (43.5)		
DLMO, decimal hrs:				
Mean (SD)	21.20 (1.36)	21.93 (1.48)	*d* = 0.51 (0.13, 1.14)	0.023 *
Median	20.5	22.00		
p25; p75	20.0; 22.0	20.75; 22.75		
N° DLMO (n, %):				
Early (<20.5 h)	7 (41,1)	2 (8.7)	V = 0.375	**0.040 ^†^**
Late (>20.5 h)	10 (58.9)	21 (91.3)		
Acrophase, decimal hrs:				
Mean (SD)	1.9 (3.7)	2 (4.1)	*d* = 0.01 (−0.64, 0.61)	0.783 *
Median	2.0	2.0		
p25; p75	23.0; 4.7	22.5, 5.0		
N° Acrophase (n, %):				
Early (<2 h)	6 (35.3)	11 (47.8)	V = 0.127	0.857 ^†^
Late (>2 h)	11 (64.7)	12 (52.2)		

Notes: Values in bold are statistically significant (*p* < 0.05). V: Cramer’s V*; d: Cohen’s d. Mann–Whitney U tests (*) or Chi-squared tests (^†^) were used to calculate differences between training groups. Effect sizes are reported as Cohen’s d (95% confidence interval) for continuous variables and Cramér’s V for categorical variables. SD = standard deviation; DLMO = dim light melatonin onset.

**Table 4:** Factors related to sleep by training group.

Variable	Training Group		Effect Size	Sig.
	Morning	Evening	(95% CI)	*p*
Training time	9:00–10:30	20:30–21:00		
Light exposure, lux	53,626	4154		
Circadian sleep pattern (mean, SD) *:				
Bedtime	0.26 (0.47)	0.63 (0.73)	d = −0.58 (−1.22, 0.06)	0.062 ^†^
Wake-up time	7.22 (0.4)	8.58 (1.54)	d = 1.13 (0.45, 1.80)	**<0.001** ^†^
Total sleep time	6.95 (0.4)	7.95 (1.31)	d = 0.97 (0.30, 1.62)	**0.014** ^†^
Chronotype (%)			**V = 0.275**	**<0.001** ^‡^
MM—moderately morning type	35.3	16.6		
N—neither type	17.6	8.3		
ME—moderately evening type	47.1	75.1		
Sleep quality (%)			**V = 0.232**	**<0.001** ^§^
Good	70.6	47.8		
Poor	29.4	52.2		
Sleepiness risk (%)			V = 0.058	0.678 ^§^
High risk	35.3	39.1		
Low risk	64.7	60.9		

Notes: Values in bold are statistically significant (*p* < 0.05); * Decimal hours. Mann–Whitney U tests (^†^), Extended Fischer tests (^‡^), or Chi-squared tests (^§^) were used to calculate differences between training groups. Effect sizes are reported as Cohen’s d (95% confidence interval) for continuous variables and Cramér’s V for categorical variables. SD = standard deviation.

## Data Availability

There are restrictions on the availability of the data for this trial, due to the signed consent agreements around data sharing, which only allow access to external researchers for studies following the project’s purposes. Requestors wishing to access the trial data used in this study can make a request to josefermar@ugr.es and mariscal@ugr.es.
